# Metabolic and Biomolecular Changes Induced by Incremental Long-Term Training in Young Thoroughbred Racehorses during First Workout Season

**DOI:** 10.3390/ani10020317

**Published:** 2020-02-18

**Authors:** Arianna Miglio, Katia Cappelli, Stefano Capomaccio, Samanta Mecocci, Maurizio Silvestrelli, Maria Teresa Antognoni

**Affiliations:** Department of Veterinary Medicine (Centro di ricerca sul Cavallo Sportivo), University of Perugia, Via San Costanzo 4, 06126 Perugia, Italy; katia.cappelli@unipg.it (K.C.); stefano.capomaccio@unipg.it (S.C.); samanta.mecocci@studenti.unipg.it (S.M.); Maurizio.silvestrelli@unipg.it (M.S.); Maria.antognoni@unipg.it (M.T.A.)

**Keywords:** metabolic modifications, long-term training, sprint exercise, biomolecular changes, immunological adaptations, Thoroughbred racehorses

## Abstract

**Simple Summary:**

Sport training leads to adaptation to physical effort that is reflected by changes in blood parameters. The Thoroughbred racehorse is a valid animal model to investigate such changes. Twenty-nine clinically healthy, two-year-old Thoroughbred racehorses were followed during their first 4-month sprint training. Blood collection was performed at rest, once a month, five times. For each sample, blood parameters were determined. Moreover, before the beginning and at the end of the experimental period, serum protein electrophoresis and genetic analysis to evaluate the expression of key genes related to inflammatory and immunity responses were performed on all samples. Significant modifications were identified compared with the beginning of training for numerous metabolites and genes related to immunity response. In conclusion, the first long-term training period induces fundamental systemic changes in untrained Thoroughbreds probably as the result of the onset of physiologic adaptation to training.

**Abstract:**

Training has a huge effect on physiological homeostasis. The Thoroughbred racehorse is a valid animal model to investigate such changes for training schedule fine-tuning. As happens in human athletes, it is hypothesized that biochemical and immune response changes and related biomolecular variations could be induced by training programs. The aim of this study was to investigate, for the first time, the long-term metabolic and biomolecular modifications in young untrained Thoroughbred racehorses in the first 4-month timeframe training period. Twenty-nine clinically healthy, untrained, two-year-old Thoroughbred racehorses were followed during their incremental 4-month sprint exercise schedule. Blood collection was performed once a month, five times (T-30, T0, T30, T60, and T90). For each sample, lactate concentration, plasma cell volume (PCV), and hematobiochemical parameters (glucose, urea, creatinine, aspartate aminotransferase (AST), γ-glutamyltransferase (GGT), alkaline phosphatase (ALP), total bilirubin (Tbil), lactate dehydrogenase (LDH), creatine kinase (CK), cholesterol, triglycerides, albumin (Alb), total proteins (TPs), phosphorus (P), calcium (Ca^2+^), magnesium (Mg), sodium (Na^+^), potassium (K^−^), and chloride (Cl)) were determined. At T-30 and T90, serum protein electrophoresis (SPE), serum amyloid A (SAA), and real-time qPCR were performed on all samples to evaluate the expression of key genes and cytokines related to inflammatory and Th2 immunity responses: Interleukin-4 (*IL-4*), Interleukin-6 (*IL-6*), Interleukin-10 (*IL-10*), Interleukin-1β (*IL-1β*), Octamer-Binding Transcription Factor 1 (*OCT1*), B-cell lymphoma/leukemia 11A (*BCL11A*). Statistical analysis was performed (ANOVA and *t* test, *p* < 0.05). Significant modifications were identified compared with T-30 for PCV, glucose, triglycerides, cholesterol, lactate, urea, creatinine, Tbil, ALP, LDH, Na^+^, K^−^, Ca^2+^, SAA, TPs, SPE, *IL*-*6, IL*-*4*, *Oct-1*, and BCL*11A*. In conclusion, the first long-term training period was found to induce fundamental systemic changes in untrained Thoroughbreds.

## 1. Introduction 

The Thoroughbred racehorse is a supreme athlete due to a high number of physiological and anatomical adaptations to exercise. Particularly, the cardiovascular system is mostly affected, although muscle remodeling and blood modifications are also reported [1]. The effects of training are related to the type, duration, and intensity of the exercise and could influence many metabolic parameters [1,2]. In the literature, there are studies focused on the modifications occurring immediately after exercise or competitions in racehorses that have demonstrated an increase of hematobiochemical parameters 5 min after exercise, which returned to basal levels after 60 min of rest [1,2,3,4,5]. Few studies have focused on long-term changes [6,7], and no studies have examined long-term variations during the first training period in a significant number of untrained Thoroughbred racehorses. We hypothesized that the first long-term training season could induce in young untrained Thoroughbred racehorses metabolic and immune response changes reflected also by modifications in the expression of related key genes, as occurs in human athletes [8,9,10]. These effects have to be deeply investigated to prevent low-performance syndromes such as overtraining, which are frequently reported to exacerbate other pathological conditions in racehorses: muscle damage (recurrent rhabdomyolysis and delayed-onset muscle soreness (DOMS)), electrolyte abnormalities, onset of blood lactate accumulation (OBLA), and exercise-induced pulmonary hemorrhage [2]. The more we know about the adaptations to training and exercise, the better the animal can compete while maintaining its welfare and allowing the expression of its excellent fitness and level of training. The importance of clinical pathology in the detection of subclinical performance-limiting disease is well described [1,2,3,4,5]. In humans, many genes have been found to be affected by exercise and they belong to pathways that play primary roles in the immune response and inflammation [8,9]. Scant data on this subject have been reported regarding racehorses [10,11]. 

Thus, the aim of this longitudinal study was to investigate the long-term metabolic modifications in untrained Thoroughbred racehorses through the evaluation of hematobiochemical parameters, serum protein electrophoresis (SPE), serum amyloid A (SAA), and related biomolecular investigations in the first 4-month time span of incremental sprint exercise to evaluate the effect of training on the health status of the animals.

## 2. Material and Methods

### 2.1. Ethical Animal Research

Sampling and sample testing were allowed by the Italian Horse Racing Board and performed under a Tissue Use Protocol approved by the Perugia University Institutional Care and Use Committee. Owner informed consent: Prior to sample collection, written owner or trainer consent was obtained for each animal.

### 2.2. Animals

Twenty-nine Thoroughbred racehorses (17 males and 12 females; two years old, weight of 350–450 kg, and height of 165–168 cm) were proven to be clinically healthy at each sample collection through a heart exam, respiratory auscultation, rectal temperature, routine hematology, and serum biochemistry analyses at rest. All horses were managed similarly with individual housing, a natural photoperiod, and natural indoor temperature. These animals were never trained for flat racing (canter and gallop) before this study. Horses were fed three times a day with 2 kg of hay supplemented with green grass, 2 kg of mixed cereal concentrate (hay pellets, corn, oats, barley, and beans), and water ad libitum. Horses were trained with the same training schedule (Table 1). Training was performed at the same time for each horse from Monday to Saturday. Each animal had one or more races after the end of the experimental period. No horses showed poor-performance syndrome during the study period or the races.

### 2.3. Sample Collection

No pharmacological treatment was administered for two weeks prior to the sample collection. The sampling activity was performed once a month, from March 2018 to July 2018, at 6:30 am before training and feeding. At T90, sample collection was performed before the race. The experimental period was divided into five times (T-30, T0, T30, T60, and T90). March (T-30) was considered as the month in which the animals started light canter; April (T0) was the first month of training simulating competitions (gallop). From April to July, training was incremental. Blood samples were collected from the jugular vein directly into Vacutainer tubes (10 mL; Terumo Corporation, BD brand; Tokyo, Japan) without additives.

### 2.4. Serum Biochemical Parameters

Immediately after blood collection, lactate (Accutrend Lactate, Roche Diagnostics, California, USA) and plasma cell volume (PCV) (Microhematocrit centrifuge, Gima, Milan, Italy)) were determined in the field. All samples were maintained at the temperature of refrigeration (4 °C) and were processed by the reference laboratory (Veterinary Clinical Pathology Laboratory, Department of Veterinary Medicine, University of Perugia) within 4 h of collection. Serum was obtained after centrifugation (3000× *g* for 10 min). No serum sample was either lipemic or hemolyzed. Selected biochemical parameters were analyzed (Hitachi 904 automated biochemistry analyzer (Boehringer Mannheim, Baden-Wurttemberg, Germany); and Seac Radim reagents (Radim diagnostics, Firenze, Italy) including glucose, triglycerides (TG), cholesterol (Chol), urea, creatinine, total bilirubin (Tbil), aspartate aminotransferase (AST), γ-glutamyltransferase (GGT), alkaline phosphatase (ALP), creatine kinase (CK), lactate dehydrogenase (LDH), phosphorus (P), magnesium (Mg), sodium (Na^+^), potassium (K^−^), chloride (Cl), calcium (Ca^2+^), total proteins (TPs), and albumin (Alb). Quality control procedures (control serum Precinorm U, Roche) were performed every 12 h with commercial QC materials, and samples were run only when the control runs passed inspection [12,13].

Serum samples were refrigerated and SAA (Equinostic EVA1, Equinostic ApS, Birkerød, Denmark) and electrophoresis (Hydragel-Hydrasis semiautomated AGE system and Hydragel 15 Protein Kit (Sebia PN 4100, Calenzano, Firenze, Italy) were performed according to the manufacturer’s recommendations within 12 h after blood collection, only for T-30 and T90 samples. The relative protein concentration within each globulin fraction was determined as the optical absorbance percentage (%), and the absolute concentration (g/L) of the same fraction was calculated from the TP concentration.

### 2.5. RT-qPCR Analyses

Total RNA was extracted from buffy coat recovered from 10 mL of total blood. Briefly, blood was centrifuged at 1800× *g* for 10 min and the white cell ring collected. Residual red blood cells were lysed with hypertonic solution (1:4) for 10 min in ice and the final suspension was centrifuged at 2000× *g*. Pellets were then resuspended in TriZol reagent (Thermoficher Scientific, Waltham, MA, USA) and placed in dry ice until extraction.

RNA from pellets was extracted using the TriZol Plus RNA purification kit (Thermoficher Scientific, Waltham, MA, USA), according to the manufacturer’s instructions, from T-30 and T90 samples. The RNA quantity and quality were assessed using the NanoDrop1000 spectrophotometer (Thermoficher Scientific, Waltham, MA, USA) and electrophoresis in a denaturing 1.2% agarose gel (Thermoficher Scientific, Waltham, MA, USA).

Total RNA (1 ug) of each sample was reverse-transcribed using the SuperScript® VILO IV™ Master Mix (Thermoficher Scientific, Waltham, MA, USA) according to the manufacturer’s recommendations. Primers for reference genes *SDHA* and *HPRT* optimal housekeeping for blood cells in horses, as well as *IL*-*6* and *IL*-*1β* genes of interest were previously determined [10,13], while genes of interest *IL*-*4*, *IL*-*10*, *BCL11A*, and *Oct-1* were designed on available sequences using the Primer-BLAST software (https://www.ncbi.nlm.nih.gov/tools/primer-blast/), trying to place them in different exons or at exon–exon junctions to avoid biases due to genomic DNA amplification. Primer sequences and accession numbers for tested genes are listed in Table 2.

The RT-qPCR reactions were carried out with 5 μL of a 10-fold-diluted cDNA template and SYBR Select MasterMix (Thermoficher Scientific, Waltham, MA, USA) for CFX (Thermoficher Scientific, Waltham, MA, USA). The amplification was performed in a CFX96 Touch instrument (BioRad, Hercules, CA, USA) with the following thermal profile: 98 °C for 3 min, then 40 cycles of 98 °C for 10 s and 58 °C for 15 s. Fluorescence data were collected at the end of the second step and, following cycling, the melting curve was determined in the range of 58–95 °C with an increment of 0.01 °C/s. Each reaction was run in triplicate with appropriate negative controls.

Data analysis was carried out with Bio-Rad CFX maestro software (ver. 4.1 BioRad, Hercules, CA, USA). To analyze gene expression stability of HKGs (Hypoxanthine-guanine phosphoribosyltransferase, *HPRT*, Succinate dehydrogenase complex, subunit A, *SDHA*), the geNorm algorithm, included with the CFX maestro software (ver. 4.1 BioRad, Hercules, CA, USA), was applied [10]. The expression ratio of the genes of interest was normalized relative to the abundance of the two reference genes using the ΔΔCq method [14].

### 2.6. Statistical Analysis

Raw data from the serum biochemical analysis were imported into R (ver. 3.4.1, https://www.r-project.org) for statistical analysis. The nlme library [15] was used to implement a linear model with the animal set as the random effect. Descriptive statistics parameters were estimated using the psych library [16]. The differences between time points were analyzed by ANOVA, setting the significance at *p* < 0.05.

RT-qPCR samples were divided into two groups (T-30 and T90) and changes in the relative gene expression between groups were calculated using a *t* test. Expression values are presented as means of fold change with the standard error using CFX maestro software (ver. 4.1- BioRad, Hercules, CA, USA).

## 3. Results

### 3.1. Serum Biochemical Parameters

**Figure 1:**
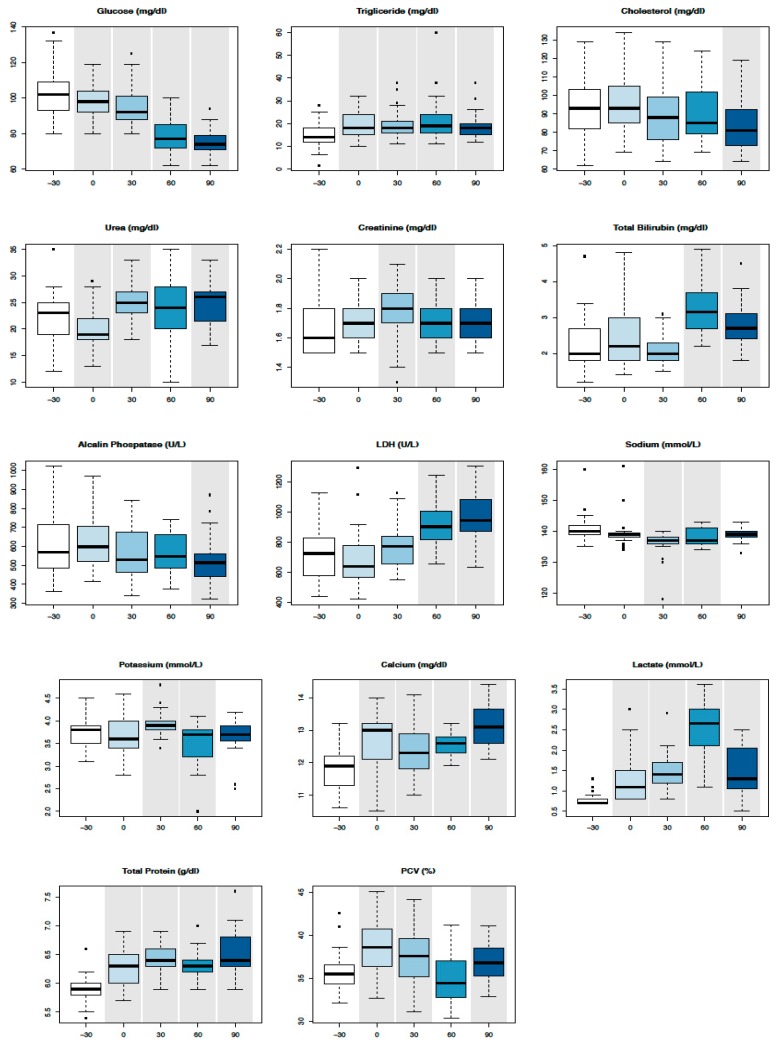
Boxplot with serum biochemical values in the time series. Circles in the plot area represent outliers. Time points that statistically differ from T-30 have a grey background.

**Figure 2:**
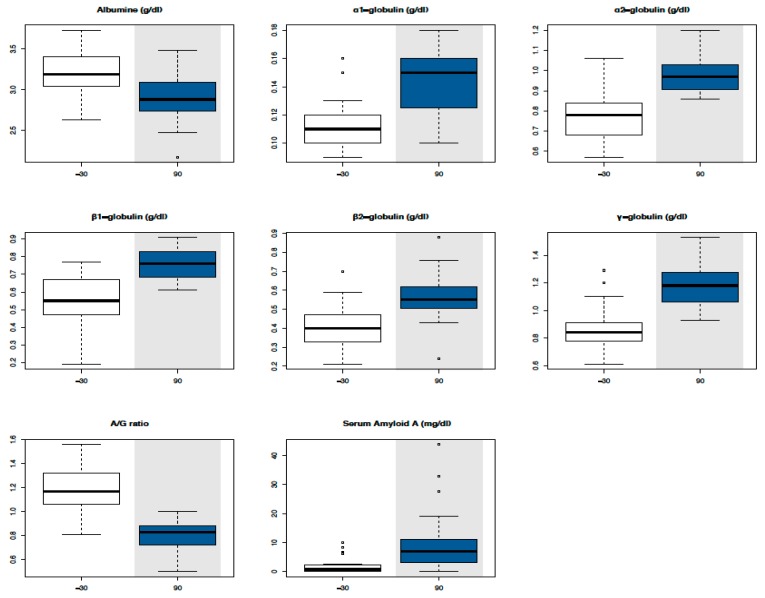
Boxplot with serum protein fraction values in the time series. Circles in the plot area represent outliers. Time points that statistically differ from T-30 have a grey background.

All results are expressed as mean values ± standard deviations in Table 3. Mean values were all within Reference Intervals (RIs) except for muscular (AST, CK, and LDH) and epathic (GGT and ALP) enzymes, lactate, and TBil in such time points where they reached levels up to twice above average. Significant changes were identified compared with T-30 for PCV (T0, T30, and T90), glucose (T0, T30, T60, and T90), triglycerides (T0, T30, T60, and T90), cholesterol (T90), urea (T0 and T30), creatinine (T30 and T60), Tbil (T60 and T90), ALP (T90), LDH (T60 and T90), Na^+^ (T30 and T60), K^−^ (T30 and T60), Ca^2+^ (T0, T30, T60, and T90), lactate (T0, T30, T60, and T90), and TPs (T0, T30, T60, and T90), as shown in the boxplots (Figure 1). SPE parameters revealed a significant increase of all globulin fractions, in particular α2-globulins and γ-globulins, and a significant decrease of the albumin/globulin (A/G) ratio at T90 (Figure 2). A significant increase of SAA concentration was evident at T90 (Figure 2).

PVC: plasma cell volume; Gluc: glucose; Trig: triglycerides; Chol: cholesterol; Urea: urea; Creat: creatinine; Tbil: total bilirubin; AST: aspartate aminotransferase; GGT: γ-glutamyltransferase; ALP: alkaline phosphatase; CK: creatine kinase; LDH: lactate dehydrogenase; P: phosphorus; Mg: magnesium; Na^+^: sodium; K^-^: potassium; CL: chloride; Ca^2+^: calcium; Lac: lactate; Alb: albumin; TP: total protein; glob: globulin; A/G ratio: albumin/globulin ratio; SAA: serum amyloid A; RIs: Reference Intervals [17,18,19].

### 3.2. RT-qPCR

**Figure 3:**
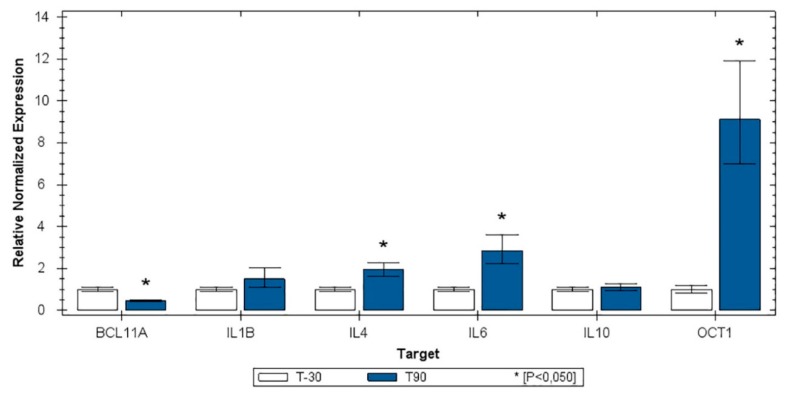
Histogram representing the relative expression values for T-30 and T90 for the investigated genes.

Figure 3 shows the relative expression distribution of *BCL11A, IL*-*10, IL*-*1β, IL*-*4, IL*-*6*, and *Oct1* transcripts of leucocytes of the 25 horses grouped by time of training (T-30, before the start of training for competitions and T90, after 4 months of incremental training simulating competitions). The expression values were normalized with two reference genes (SDHA and HPRT). Reference genes showed relatively high stability, with an M value below the accepted threshold [10]. Significant differences in gene expression between groups were found for *IL*-*4* and *IL*-*6*, whereas *IL*-*10* and *IL*-*1β* were not modulated. The *Oct-1* gene was strongly upregulated in T90 samples, whereas *BCL11A* was downregulated (Figure 3).

## 4. Discussion

There have been few advances in the use of clinical pathology as an indicator of performance potential in racehorses in the past 30 years. This is the first study presenting the changes in blood parameters and related gene expression profiling of Thoroughbred racehorses monitored for 4 months in their first training season. 

According to the literature, exercise has a powerful action on metabolism, which is influenced by the intensity and duration of the effort both in humans and racehorses [1,2,3,4,20,21,22,23]. Our results showed a significant and progressive decrease in glycaemia that remained within the RIs. It is well known, both in humans and horses, that gene expression of glucose transporter type 4 (GLUT-4) is increased after exercise, which determines the translocation of GLUT-4 from intracellular sites to muscle sarcolemma [21,24]. Indeed, over the course of long-term training, both insulin sensitivity and glucose uptake are enhanced, producing a reduction in blood glucose levels even if remaining in physiological values [22]. Long-term training provokes a large shift in substrate utilization [20]. Exercise in racehorses has been demonstrated to induce a reduced level of carbohydrates and an increased level of lipids as an energy substrate. Lipolysis in muscle and adipose tissue, as well as hepatic synthesis of triglycerides are enhanced due to the increased oxidative capacity for fat utilization during training [21,22,25]. Lipolysis, other than glycogenolysis and gluconeogenesis, is assumed to be stimulated by catecholamines and other catabolic hormones related to stress (insulin and cortisol) and is thus reported to affect glucose and lipid metabolism and provoke an exercise-induced transient hyperlipemia [23,25]. Regarding Thoroughbreds, it has been demonstrated that racehorses reach higher levels of circulating TG (2× greater) and cholesterol (20%) at rest after 8 weeks of training compared with riding horses [23]. Our data are, in part, in line with these metabolic adjustments. They show, for the first time in Thoroughbreds, a mild long-lasting increase in triglycerides during long-term, high-sprint-intensity training. Probably, as happens in endurance horses after long distances, TG play a crucial role in replenishing the intramuscular fat stores depleted in the course of repeated training sessions [3,23]. 

From a bimolecular level, in humans, it has been demonstrated that glycogen depletion in working muscles stimulates the production of myokines (i.e., cytokines produced from myocytes) in response to muscle contractions [26]. Receptors for myokines are found in muscle, fat, liver, pancreas, bone, heart, immune, and brain cells. Thus, myokines have multiple functions and they are involved in exercise-associated metabolic changes following training adaptation [26]. *IL*-*6* is a cytokine believed to play a dominant role in this context and a network of signaling cascades, including the Ca^2+^/NFAT and glycogen/p38 MAPK pathways, regulating its expression. It has pleiotropic effects, including both inflammatory and anti-inflammatory functions, hepatic lipolysis, immuno-modulation, and acute phase response. It has a beneficial impact on health and body functioning when elevated in response to physical exercise [11,26,27]. In our study, biomolecular results revealed the upregulation of the *IL*-*6* gene*,* as occurs in trained endurance horses compared with untrained [28]. In particular, during exercise, *IL*-*6* has autocrine, paracrine, and/or endocrine effects, such as to mobilize extracellular substrates and augment substrate delivery [26,27]. We found a slight progressive decrease in cholesterol levels with a significant lower mean value at T90. This result disagrees with that above reported on Thoroughbred racehorses [23], probably because we tested horses that had never been trained before during a longer period of training. On the other hand, this result agrees with those reported about humans over the course of long-term training, as a consequence of the upregulation of *IL*-*6* [27], as our data also confirmed (Figure 3). The increasing liver production of several enzymes and proteins (such as SAA and globulins) that enhance the reverse cholesterol transport system, thus altering the rates at which cholesterol is synthesized, transported, and cleared from the blood, could also contribute to its decrease [26,27].

Our data revealed a significant increase in lactate concentrations at all time points, with most of the values remaining within the RIs. During the course of high-intensity sprint exercise, ATP production is due to both aerobic (70%) and anaerobic metabolism. At a very high speed level, Thoroughbreds use mostly the anaerobic supplies and an increase in blood lactate concentrations has been reported in trained horses after exercise [29]. In our study, the highest values at rest were identified at T60, probably due to the beginning of the maximal sprint exercise levels. Often, lactate has been argued to be a molecule mainly responsible of fatigue, but new data suggest this molecule may be an efficient energy source and plays an important role in exercise-mediated adaptation [30]. Thoroughbred horses have a higher capacity to sustain maximal exercise than other breeds, and it is attributable to their physiological characteristics, such as a high maximal oxygen uptake (VO_2_ max), large stores of muscle glycogen, and high glycolytic and oxidative capacity, thus resulting in a high capacity for lactate production and oxidation [29,30]. 

Calcium levels were also increased at all times compared with T-30, although the mean values were within the RIs. In human medicine, it has been described that increases in lactate levels due to high speed exercise produce metabolic acidosis and, therefore, increase values of ionized calcium [24,31]. Recent progress explains the rise of serum calcium levels with the increase of training in human athletes due to the lower Ca^2+^ uptake of their RBCs, which contain less free Ca^2+^ at rest than the cells of untrained controls [32].

Concerning muscle-derived enzymes (AST, CK, and LDH), mean activities were within the RIs for two-year-old Thoroughbreds in training [16] and were about 2-fold higher than the RIs for adult Thoroughbreds [17,18,19]. LDH activity showed a significant increase, slightly over the RI of two-year-old Thoroughbreds, at T60 and T90. The literature reports unexplained increases of muscle enzymes of young horses at the beginning of the training season due to musculoskeletal adaptation to exercise conditioning [1,2,7]. This might reflect increases in mitochondrial membrane permeability or slight muscle damage, which may provoke DOMS or rhabdomyolysis due to increased training intensity [1,2,7]. 

The mean activities of liver enzymes (ALP and GGT) were within the RIs for two-year-old Thoroughbreds in training [17] and about 2-fold higher than the RIs of adult Thoroughbreds [17,18,19]. ALP showed a significant decrease at T90. These results agree with those reported in humans and horses that indicate activities decreased for ALP and elevated GGT during training [1,2,22]. ALP is an enzyme found primarily in liver and bone, and in humans, lower resting levels could be a consequence of strain on the bone structure [22]. The weight of these animals together with the demands induced by gallop could also cause lower levels in horses. GGT activity is positively correlated with cumulative days in training and the values identified in our study were lower than the limit (100 UI/L) associated with poor performance [21]. Slightly high mean values of GGT during exercise seem to be related to a physiological process useful for reducing oxidative stress by breaking down extracellular glutathione and making its components available to cells for repair [1,2,21,22].

Tbil was slightly higher than the RIs and showed a significant increase at T60 and T90. Tbil has been reported to increase after a long training period in horses due to accelerated RBC breakdown stimulated by exercise conditions, such as muscle contraction and impact, and/or reduced caloric intake [1,2]. Interestingly, the higher mean values were reached at T60, with the increased workload, when the PCV mean level was slightly decreased compared with T-30.

Urea and creatinine were within RIs even if they showed a significant elevation compared with T-30. This could have been due to the extensive fluid loss in the sweat, the reduction of renal flow, and the muscle damage responsible for the increased protein catabolism [1,2,3,4].

Electrolytes remained within RIs in all time points, but significant changes were identified for sodium and potassium, probably due to sodium perspiration due to high-speed exercise and dietary restriction, and for the release of potassium from exercising muscles to extracellular spaces [1,2,3,4].

In the literature, few works describe the effects of exercise on serum proteins in racehorses and they show the modifications induced immediately after training and racing [5,32,33,34]. To our knowledge, this is the first study showing the effect of long-term training on TPs and SPE in untrained racehorses. TP concentrations were within RIs even if they showed a significant increase in all time points compared with T-30, as happens in untrained endurance horses after the first training season [35]. Since PCV showed significantly increased levels during the experimental period (T0, T30, and T90), even if within the RI for two-year-old Thoroughbreds, the correlation between TPs and PCV was tested to exclude dehydration and it was found to be considerably low. Thus, to evaluate the changes of each protein fraction, SPE was performed. Alb and the A/G ratio showed a significant decrease, whereas all globulin fractions showed a significant increase at T90, even if all within RIs with the exception of α2- and γ-globulins, the concentrations of which were slightly over the RI for two-year-old Thoroughbreds.

SAA, a major acute phase protein in horses migrating in the α2 protein fraction [35], was evaluated for the first time in untrained racehorses after the first long training period (3 months) and before the races. SAA in inexperienced Thoroughbreds was significantly increased after a long training period (approximately 10-fold), even if within RIs. On the contrary, in untrained endurance horses, the increase of SAA concentrations has been observed only after competition and not during the first seven-month training program [36]. This difference could be explained by the fact that exercise-induced acute phase response seems to be evident in the course of a high load of exercise, inducing microinjuries and/or glycogen depletion in working muscle, which stimulates cytokine production [36]. Indeed, *IL*-*6* and *IL*-*4* (the transcription of the key Th2 response cytokine genes) were upregulated in T90, and both promote the production of SAA in the liver [26,36]. We cannot completely exclude the presence of subclinical conditions (infectious or inflammatory) responsible for slight increases of SAA, but at the same time of blood sampling, each animal had no clinical signs and white blood cell counts were within the physiological range (data not reported). 

In horses, an anti-inflammatory state has been reported both in race [11] and endurance training [37]. In humans and horses, IL-6, in its role as a myokine, has been related to an anti-inflammatory function by inhibiting the expression of proinflammatory cytokines, such as *IL*-*1β* [8,10,26], which, indeed, was not modulated in our study. Moreover, the upregulation of *IL*-*6* secondary to an inflammatory status is generally followed by the stimulation of the anti-inflammatory cytokine *IL-10* [9,10,26], which, on the contrary, was not modulated in T90. Our study supports the hypothesis that a slight increase of SAA during long-term training in unexperienced racehorses probably does not indicate inflammation but rather the physiological response to heavy effort at the beginning of their carrier [36]. It may be interpreted as either an indication of harmful training or the normal onset of adaptation to the training routine inducing the stimulation of a systematic reaction [33]. The evaluation of SAA levels after exercise and racing may be helpful for detecting pathology in young racehorses in training. 

Probably, the *IL-6* upregulation in trained Thoroughbred racehorses could be fundamental for the adaptation to exercise, leading to a reduced inflammatory response rather than to metabolic changes and immunomodulation.

β2 and γ fractions are generally increased in the course of antigenic stimulations that induce immunoglobulin production (IgM-Immunoglobulin M and IgG-Immunoglobulin G) [6,34]. In humans, it is known that the increase in globulins at rest, especially immunoglobulins, could be a result of long-term regular exercise, which produces immunological stimulation due to the increased secretion of catecholamine, neuropeptides, and myokines and the decreased basal levels of cortisol [38]. There is a lack of data on this topic in racehorses. To deeply investigate the reliability of β- and γ-globulins increasing during training, we analyzed the expression of key transcription factors and cytokines related to Th2 immunity response. As shown in humans, our results demonstrated that the key inducers of γ-globulin expression—*Oct-1* transcription factor (an Ig gene promoter) [39], *IL*-*4* (promoting Th2 differentiation) [38], and *IL*-*6*—were strongly upregulated in T90, whereas BCL*11A* (a repressor of γ-globulin gene expression) [40] was downregulated. These results are indicative of increased exercise-induced immunoglobulin synthesis and suggest that long-term training may exert a conditioning effect on gene expression at rest in Thoroughbreds.

## 5. Conclusions

Regular long-term training has a significant effect on metabolism, acute phase response, and the immune system in young Thoroughbred racehorses during the first workout season. Probably, these modifications are the result of the onset of physiological adaptations to the training routine by inducing the stimulation of systematic reactions. Resting values of the serum parameters and the biomolecular tests analyzed could be useful to assess the status of an athlete and the degree of training adaptability, as well as to avoid poor performance. Future studies have a particular focus on myokine research in response to long-term exercise in racehorses.

## Figures and Tables

**Table 1:** Standard daily training program completed by each horse involved in the study. Speeds: Walk: 100 m/min, Trot: 200 m/min, Canter: 350 m/min, and Gallop: 1000 m/min (min: minutes).

February	March (T-30)	April (T0)	May (T30)	June (T60)	July (T90)
15 min Walk 10 min Trot Rest 10 min Trot Walk	15 min Walk 10 min Trot 3 min Canter	15 min Walk 10 min Trot 6 min Canter every Tuesday: 1 min Gallop	15 min Walk 10 min Trot 6 min Canter every Tuesday: 2 min Gallop	15 min Walk 10 min Trot 6 min Canter every Tuesday: 3 min Gallop	15 min Walk 10 min Trot 6 min Canter every Tuesday: 4 min Gallop

**Table 2:** Genes evaluated in this study: Interleukin-4 (*IL-4*), Interleukin-6 (*IL-6*), Interleukin-10 (*IL-10*), Interleukin-1β (*IL-1β*), Octamer-Binding Transcription Factor 1 (*OCT1*), B-cell lymphoma/leukemia 11A (*BCL11A*) Hypoxanthine-guanine phosphoribosyltransferase, (*HPRT*), Succinate dehydrogenase complex, subunit A (*SDHA*). Relative primer pairs used.

Gene Name	Accession ID	Amplicon Length (bp)	Primer Forward	Primer Reverse	Reference
*IL-4*	NM_001082519.1	75	AAGAATGCCTGAGCGGACTG	TGGCTTCATTCACAGTACAGCA	This work
*IL-10*	XM_014739408.1	107	TTCAGCAGGGTGAAGACTTTCT	AAGGCTTGGCAACCCAGGTA	This work
*Oct1*	XM_023640479.1	165	GATTGAGGGCTTGAACCGC	ACCAAACACGAATCACCTCC	This work
*BCL11A*	XM_023619062.1	87	TTTGCCCCAAACAGGAACAC	ATGCACTGGTGAATGGCTGT	This work
*IL-6*	NM_001082496	98	TCAAGGGTGAAAAGGAAAACATC	GGTGGTTACTTCTGGATTCTTC	[8]
*IL-1β*	NM_001082526	135	AGAACCTGTACCTGTCTTGTG	CGTTGCCCTTGATTTCCATC	[8]
*HPRT1*	AY372182	121	AATTATGGACAGGACTGAACGG	ATAATCCAGCAGGTCAGCAAAG	[9]
*SDHA*	DQ402987	91	GAGGAATGGTCTGGAATACTG	GCCTCTGCTCCATAAATCG	[9]

**Table 3:** Means ± standard deviations of biochemical and electrophoretic parameters. Parameters significantly modified compared with T-30 are in bold.

Parameters	T-30	T0	T30	T60	T90	RIs of Two-Year-Old Thoroughbred Horses in Training [17,18,19]	RIs of Adult Thoroughbred Horses at Rest [17,18,19]
PCV (%)	35 ± 2.5	**38 ± 3.1**	**37 ± 3.3**	35 ± 3.09	**36 ± 2.2**	34–45	38–50
Gluc (mg/dL)	104 ± 14.6	**98 ± 9.3**	**95 ± 11.6**	**78 ± 9.5**	**75 ± 6.9**	61–106	75–115
TG (mg/dL)	15 ± 5.5	**19 ± 5.8**	**19 ± 6.9**	**22 ± 10.6**	**19 ± 5.8**	23–230	4–44
Chol (mg/dL)	92.2 ± 16.0	94.8 ± 17.1	90.1 ± 17.2	90.5 ± 15.3	**83.2 ± 14.4**	77–128	75–150
Urea (mg/dL)	22 ± 4.7	**19 ± 4.2**	**25 ± 3.9**	23 ± 5.8	**24 ± 4.0**	11–20	21.4–51.4
Creat (mg/dL)	1.6 ± 0.1	1.6 ± 0.1	**1.7 ± 0.1**	**1.7 ± 0.1**	1.7 ± 0.1	1.2–1.8	1.2–1.9
Tbil (mg/dL)	2.2 ± 0.7	2.5 ± 0.8	2.064 ± 0.4	**3.2 ± 0.6**	**2.7 ± 0.6**	0.8–2.3	1–2
AST (IU/L)	501 ± 211	477 ± 262	474 ± 221	489 ± 213	453 ± 148	308–820	243–327
GGT (IU/L)	22 ± 10.6	27 ± 18.0	23 ± 10.1	23 ± 14.0	22 ± 7.8	12–40	4.3–13.4
ALP (IU/L)	591 ± 149	619 ± 133	569 ± 141	561 ± 99	**520 ± 129**	293–672	143–395
CK (IU/L)	364 ± 176	412 ± 387	337 ± 137	347 ± 156	392 ± 196	166–572	76–154
LDH (IU/L)	735 ± 183	686 ± 195	777 ± 146	**920 ± 146**	**953 ± 168**	569–917	477–813
P (mg/dL)	4.8 ± 0.7	4.893 ± 0.5	4.9 ± 0.7	4.6 ± 0.8	4.9 ± 0.7	3.4–5.0	3.1–5.6
Mg (mg/dL)	1.7 ± 1.1	1.9 ± 0.0	2.0 ± 0.1	1.9 ± 0.3	1.9 ± 0.1	1.5–2.2	2.2–2.8
Na (mmol/L)	140 ± 4.4	139 ± 4.9	**136 ± 4.4**	**137 ± 2.7**	139 ± 1.9	130–142	132–146
K (mmol/L)	3.7 ± 0.3	3.6 ± 0.4	**3.9 ± 0.2**	**3.5 ± 0.5**	3.6 ± 0.4	2.8–4.2	2.4–4.7
CL (mmol/L)	98.7 ± 2.0	99.5 ± 8.0	98.4 ± 1.2	99.6 ± 1.2	99.7 ± 1.8	99–109	99–109
Ca (mg/dL)	11.8 ± 0.6	**12.5 ± 0.9**	**12.3 ± 0.8**	**12.5 ± 0.3**	**13.1 ± 0.6**	11.6–13.2	11.2–13.6
Lac (mmol/L)	0.7 ± 0.1	**1.2 ± 0.5**	**1.4 ± 0.4**	**2.6 ± 0.6**	**1.4 ± 0.5**	1.11–1.78	1.11–1.78
Alb (g/dL)	3.8 ± 0.2	3.9 ± 0.2	3.8 ± 0.2	3.8 ± 0.2	**3.5 ± 0.2**	3.5–3.9	2.6–3.7
TP (g/dL)	5.9 ± 0.2	**6.2 ± 0.3**	**6.4 ± 0.2**	**6.3 ± 0.2**	**6.5 ± 0.3**	5.9–6.6	5.2–7.9
α1-glob (g/dL)	0.11 ± 0.02	**-**	**-**	**-**	**0.14 ± 0.31**	0.06–0.14	0.06–0.7
α2-glob (g/dL)	0.77 ± 0.11	**-**	**-**	**-**	**0.97 ± 0.02**	0.54–0.78	0.31–1.31
β1-glob (g/dL)	0.55 ± 0.14	**-**	**-**	**-**	**0.75 ± 0.08**	0.57–0.85	0.4–1.58
β2-glob (g/dL)	0.39 ± 0.10	**-**	**-**	**-**	**0.56 ± 0.12**	0.18–0.68	0.29–0.89
γ-glob (g/dL)	0.87 ± 0.14	**-**	**-**	**-**	**1.18 ± 0.14**	0.37–0.82	0.55–1.9
A/G ratio	1.12 ± 0.2				**0.8 ± 0.1**	0.6–1.46	0.6–1.46
SAA(mg/L)	1.9 ± 2.9				**10.2 ± 11.1**	0.0–20.0	0.0–20.0

## Abbreviations

PCV	plasma cell volume
TG	triglycerides
Chol	cholesterol
AST	aspartate aminotransferase
GGT	γ-glutamyltransferase
ALP	alkaline phosphatase
Tbil	total bilirubin
LDH	lactate dehydrogenase
CK	creatine kinase
Alb	albumin
TPs	total proteins
P	phosphorus
Ca^2+^	calcium
Mg	magnesium
Na^+^	sodium
K^−^	potassium
Cl	chloride
SPE	serum protein electrophoresis
SAA	serum amyloid A
RT-qPCR	real-time qPCR
IL-4	Interleukin-4
IL-6	Interleukin-6
IL-10	Interleukin-10
IL-1β	Interleukin-1β
OCT1	Octamer-Binding Transcription Factor 1, https://www.genecards.org/cgi-bin/carddisp.pl?gene=POU2F1
BCL11A	https://www.uniprot.org/uniprot/Q9H165
